# RNA demethylase ALKBH5 regulates cell cycle progression in DNA damage response

**DOI:** 10.1038/s41598-025-01207-8

**Published:** 2025-05-08

**Authors:** Bo Gao, Haitao Pan, Xiaoling Zhou, Lei Yu, Yunyi Gao, Tao Zhang, Xiangwei Gao, Jingyu Hou

**Affiliations:** 1 Shaoxing Maternity and Child Health Care Hospital, Shaoxing, 312000 China; 2 Environmental Medicine, School of Public Health, Zhejiang University School of Medicine, Hangzhou, 310058 China

**Keywords:** ALKBH5, DNA damage response, m6A, mRNA stability, Cell cycle, Cell biology, Molecular biology

## Abstract

RNA N6-methyladenosine (m6A) modification plays a crucial role in the DNA damage response, while the detailed mechanisms remain to be explored. In this study, we report the involvement of the m6A demethylase ALKBH5 in X-ray-induced DNA damage response. Depletion of ALKBH5 reduces X-ray-induced DNA damage, induces G2/M phase arrest and reduces cell apoptosis. RNA sequencing and m6A sequencing analysis reveal that ALKBH5 removes m6A modifications from its target mRNAs and suppresses their expression. A subset of mRNAs encoding cyclin dependent kinase inhibitors, such as CDKN1A and CDKN2B, show increased stability and expression upon ALKBH5 knockdown. Subsequently, the upregulation of CDKN1A and CDKN2B contributes to G2/M phase arrest to facilitate DNA repair. Our findings unveil the epigenetic regulation of cell cycle checkpoint by ALKBH5 in X-ray-induced DNA damage, offering potential targets for DNA damage-based therapy for cancers.

**Supplementary Information:**

The online version contains supplementary material available at 10.1038/s41598-025-01207-8.

## Introduction

X-ray represents a highly energetic form of ionizing radiation extensively employed in medical diagnostics and scientific research, leading to increased exposure and growing concerns over the associated health risks^1–5^. DNA serves as the primary target of X-ray radiation^6^, and this radiation induces various forms of damage, with DNA double-strand breaks (DSBs) being the most severe. Unrepaired DSBs can lead to gene mutations and functional disorders. The DNA damage response (DDR) is a cascade that senses, amplifies, and transmits DNA damage signals^7,8^. It integrates key biological processes, including cell cycle regulation, DNA repair, and apoptosis, all of which are critical for preserving genomic stability^7,8^.

Cell cycle checkpoints are essential mechanisms for monitoring and repairing DNA damage^9,10^. In interphase, cells must pass through two checkpoints: one that allows progression from G1 (G1 checkpoint) to chromosome replication (S phase), and another that allows transition from G2 (G2 checkpoint) to mitosis (M phase)^9^. Specific cyclin and cyclin-dependent kinase (CDK) complexes are sequentially activated during different phases of the cell cycle, phosphorylating their corresponding substrates to ensure the orderly progression of cell cycle^11,12^. When DNA damage occurs, kinases including ATM/ATR can directly phosphorylate p53, or indirectly lead to its phosphorylation by activating checkpoint kinases 1 and 2 (CHK1/2), which increases p53 expression and transcriptional activity^13,14^. Activated p53, as the master regulator, can halt the cell cycle at the G1 or G2 phase via the Cdk2/Cyclin E and Cdk1/Cyclin B pathways, providing sufficient time for DNA repair^15–17^. Once DNA damage cannot be repaired, p53 triggers apoptosis to prevent the replication of damaged chromosome. While the cell cycle is halted, DNA repair pathways work simultaneously and alleviate stress, which is crucial for maintaining genomic stability. Defects in the cell cycle checkpoints can lead to chromosome damage and the erroneous transmission of genetic information.

N6-methyladenosine (m6A) is the most abundant internal modification in eukaryotic mRNAs and is widely recognized for its role in gene expression regulation^18,19^. The m6A modification is dynamically reversible, which is regulated by methyltransferases (‘writers’) and demethylases (‘erasers’). The writers, known as the methyltransferase complex (MTC), consist of core components METTL3, METTL14, WTAP, and auxiliary factors that facilitate the deposition of m6A on mRNAs^20,21^. The removal of m6A modifications depends on ‘erasers’, including FTO and ALKBH5^22–24^. The biological functions of m6A rely on different ‘reader’ proteins, including the YTH domain family proteins (YTHDF1-3 and YTHDC1-2) and the insulin-like growth factor 2 mRNA-binding protein (IGF2BP) family, which regulate mRNA metabolism such as splicing, transport, translation, and degradation^25–27^.

Recent studies have shown that RNA m6A machinery plays crucial roles in the DDR. METTL3 is rapidly recruited to UV-induced cyclobutene pyrimidine dimers (CPDs), promoting Poly(A) RNA m6A deposition and facilitating the recruitment of DNA polymerase κ (Pol κ) to remove CPDs, thereby enhancing cellular resistance to UV damage^28^. METTL3-mediated m6A modifications also impact the repair of double-strand breaks (DSBs). METTL3 is activated by ATM phosphorylation and localized at DSB sites, promoting m6A modification of nascent RNA^29^. The accumulation of numerous DNA-RNA hybrids at DNA damage sites recruits RAD51 and BRCA1 to initiate homologous recombination (HR) repair^29^. Studies have also suggested a role of ALKBH5 in the DDR. reactive oxygen species promote ALKBH5 SUMOylation, which inhibits ALKBH5 m6A demethylase activity. This leads to the rapid and efficient induction of thousands of genes involved in a variety of biological processes including DNA damage repair^30^. Another study showed that ALKBH5 increases the stability of HMGB1 mRNA by removing its m6A modification, thereby promoting the activation of the STING signaling pathway and facilitating radiation-induced hepatocyte apoptosis^31^. However, the precise role of ALKBH5 in the DDR remains unclear and requires further exploration.

In this study, we report a role of m6A demethylase ALKBH5 in the DDR triggered by X-ray radiation. ALKBH5 removes the m6A modification from cyclin dependent kinase inhibitors such as CDKN1A and CDKN2B, reducing their stability and expression. This results in cell cycle progression and DNA damage repair, ultimately impacting the DNA damage outcomes of cells. Understanding the epigenetic regulation of cell cycle checkpoints by ALKBH5 offers new insights for X-ray-induced DNA damage response.

## Results

### ALKBH5 depletion reduces X-ray-induced DNA damage

**Fig. 1:**
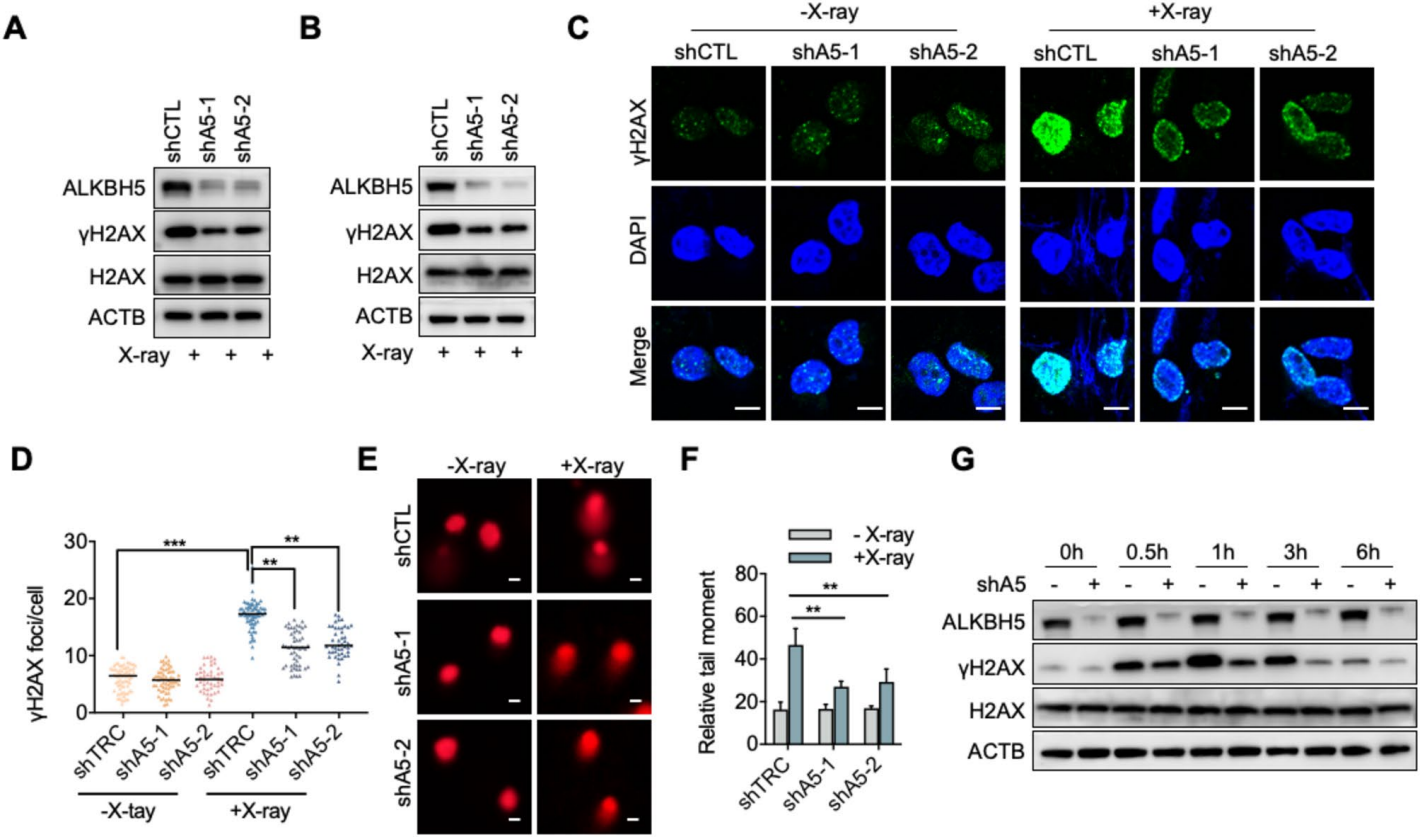
Knockdown of ALKBH5 reduces X-ray-induced DNA damage. (**A**) Immunoblotting analysis of γH2AX level in HEK293T cells following X-ray exposure (10 Gy). Original blots are presented separately. (**B**) Immunoblotting analysis of γH2AX level in HCT116 cells following X-ray exposure (10 Gy). Original blots are presented separately. (**C**) Immunofluorescence detection of γH2AX foci formation. Scale bars represent 10 μm. (**D**) Quantification of the number of γH2AX foci per cell. The values are indicated as Mean ± SD. Data were compared statistically using the two-tailed Student’s t-test. (**, *p* < 0.01; ***, *p* < 0.001). (**E**) Comet assay for detecting DNA damage under the same conditions as (C). Scale bars represent 20 μm. (**F**) Quantification of (E). The relative tail moments of HEK293T cells (*n* > 50 cells). The values obtained are indicated as Mean ± SD. Data were compared statistically using the two-tailed Student’s t-test. (**, *p* < 0.01). (**G**) Time-course analysis of γH2AX level in HEK293T cells following X-ray exposure. Original blots are presented separately.

To investigate the role of m6A ‘eraser’ ALKBH5 in X-ray-induced DNA damage, we constructed two ALKBH5 knockdown HEK293T cell lines using short hairpin RNAs (shRNAs). The cells were exposed to 10 Gy of X-ray irradiation (Fig. 1A), a dose that is sufficient to induce DNA damage. Compared to the control, ALKBH5 knockdown significantly reduced γH2AX formation after X-ray irradiation, suggesting that ALKBH5 may play a crucial role in the X-ray irradiation induced DDR (Fig. 1A). ALKBH5 also reduced γH2AX formation after X-ray irradiation in HCT116 colorectal cancer cells (Fig. 1B). Immunofluorescence revealed that both control and ALKBH5 knockdown cells had few γH2AX foci under normal conditions (Fig. 1C). Following X-ray irradiation, the formation and intensity of γH2AX foci markedly increased in the nucleus (Fig. 1C,D). To assess the extent of DNA damage, a neutral comet assay was performed. The neutral comet assay results showed pronounced comet tails in cells after X-ray irradiation, while ALKBH5 knockdown significantly reduced the olive tail moment and area (Fig. 1E,F). To distinguish whether γH2AX foci reduction in ALKBH5 knockdown cells reflects diminished DNA damage formation or faster resolution of DNA damage, we performed time-course analyses of γH2AX formation. The data showed that both control and ALKBH5 knockdown cells reached numerous and comparable γH2AX foci at 0.5 h. By 3 h, knockdown cells resolved most of γH2AX foci, whereas control cells retain a fraction. The time-course experiment collectively supported that the observed γH2AX reduction at 1 h is not due to diminished damage but accelerated DNA repair enabled by ALKBH5 (Fig. 1G). Altogether, these findings suggest that ALKBH5 knockdown mitigates X-ray-induced DNA damage.

### ALKBH5 depletion enhances the efficiency of DSB repair

**Fig. 2:**
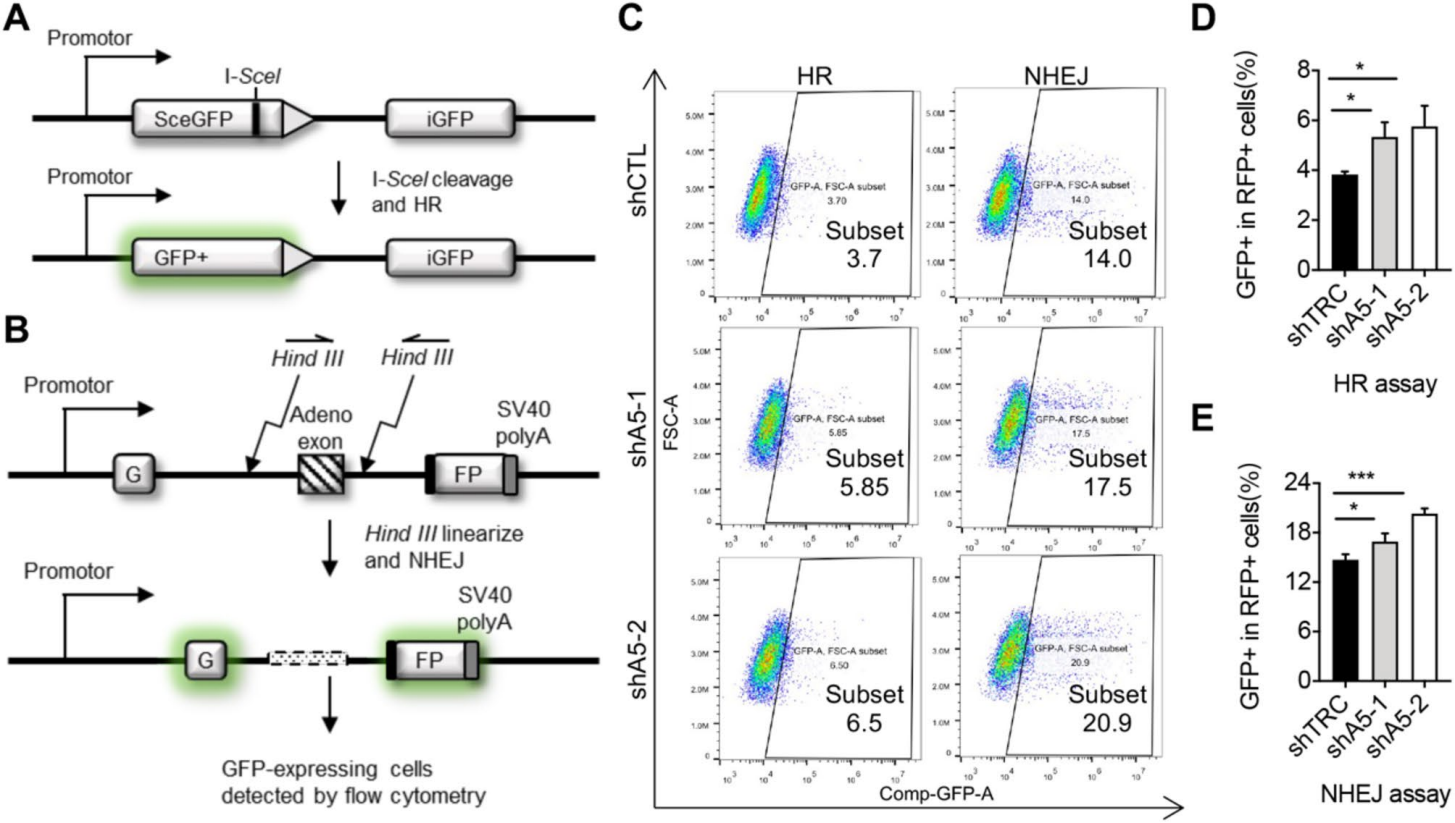
Knockdown of ALKBH5 enhances the efficiency of DSB repair post X-ray exposure. (**A**) Schematic of the reporter system for the analysis of HR-mediated DSB repair. (**B**) Schematic of the reporter system for the analysis of NHEJ-mediated DSB repair. (**C**) Landscape of GFP + cells determined by FACS analysis. Cells transfected with HR reporter or NHEJ reporter together with mCherry-expressing internal control plasmid were treated with X-ray (10 Gy). The expression of GFP and Cherry were determined by flowcytometry after 48 h. The proportion of GFP + cells in Cherry + cells was obtained as a relative frequency. The framed area indicates the percentage of GFP+/Cherry+. (**D**) Quantification of the frequency of HR-mediated DSBR in control and ALKBH5-KD cells. The values are indicated as Mean ± SD. Data were compared statistically using the two-tailed Student’s t-test. (*, *p* < 0.05). (**E**) Quantification of the frequency of NHEJ-mediated DSBR in in control and ALKBH5-KD cells. The values are indicated as Mean ± SD. Data were compared statistically using the two-tailed Student’s t-test. (*, *p* < 0.05; ***, *p* < 0.001).

DSBs are the most common and severe form of X-ray-induced DNA damage, primarily repaired through two main pathways: high-fidelity homologous recombination (HR) and error-prone non-homologous end joining (NHEJ)^32–34^. To explore the impact of ALKBH5 on these repair pathways, we employed fluorescence reporter systems. The HR reporter plasmid utilizes the I-SceI endonuclease to introduce DSBs at the green fluorescent protein (Sce-GFP) gene (Fig. 2A). The iGFP sequence on the same vector serves as a homologous template for DNA repair (Fig. 2A). The NHEJ reporter plasmid is constructed by cleaving the GFP gene with HindIII, generating two broken ends (Fig. 2B). Successful repair leads to the rejoining of these ends, resulting in GFP fluorescence (Fig. 2B). We also used a red fluorescent p-Cherry plasmid as an internal control for transfection efficiency. Both reporter systems were co-transfected into control and ALKBH5-depleted cells. After 48 h of culture, we harvested the cells and measured the fluorescence signals using flow cytometry (Fig. 2C). After correcting for transfection efficiency using red fluorescence, our results showed that ALKBH5-depleted cells showed a significant increase in the ratio of GFP-positive cells (GFP+) compared to control cells (Fig. 2C–E). This indicates that knockdown of ALKBH5 promotes the repair of DNA double-strand breaks mediated by both HR and NHEJ pathways, thereby reducing X-ray-induced DNA damage.

### ALKBH5 depletion increases m6A modification and gene expression under X-ray radiation

**Fig. 3:**
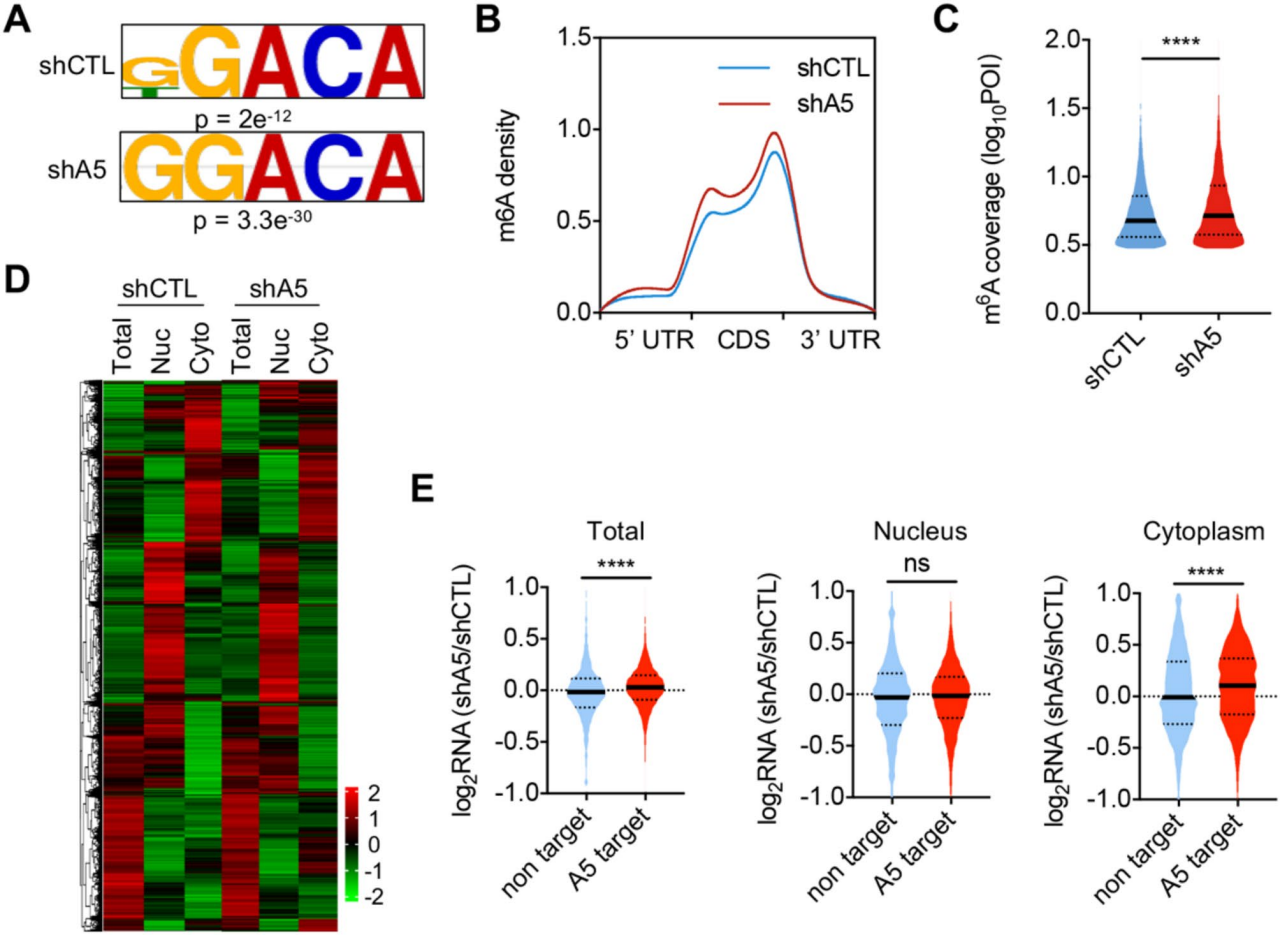
Knockdown of ALKBH5 increases m6A modification and expression levels of mRNAs. (**A**) Consensus motifs of m6A sites in control and ALKBH5-KD cells exposed to X-rays. (**B**) Metagene plot of mRNA m6A peak distribution in control and ALKBH5-KD cells following X-ray irradiation. (**C**) Violin plot displaying RNA m6A abundance in the control and ALKBH5 knockdown cells after X-ray exposure. POI, peak over input. The upper and lower quartiles, as well as the median, are indicated for each group. Statistical significance was determined using the Mann-Whitney test. (****, *p* < 0.0001). (**D**) Heatmap of RNA expression in whole cell, nuclei, and cytoplasm of control and ALKBH5 knockdown cells after X-ray exposure. (**E**) Violin plot displaying RNA expression changes of ALKBH5 target and non target after ALKBH5 knockdown following X-ray exposure. The upper and lower quartiles, as well as the median, are indicated for each group. Statistical significance was determined using the Mann-Whitney test. (ns, not significant; ****, *p* < 0.0001).

ALKBH5 acts as an m6A ‘eraser’. To further explore its role in the DDR, we performed m6A-seq on both control and ALKBH5 knockdown cells after X-ray exposure. Consistent with existing studies, the m6A sequencing results identified m6A peaks that are characterized by the canonical RRACH motif (Fig. 3A). The distribution of m6A in transcripts was enriched around stop codons (Fig. 3B), and ALKBH5 knockdown significantly increased the abundance and coverage of m6A peaks (Fig. 3B,C). ALKBH5 catalyzes the demethylation of nuclear RNAs, primarily mRNAs, thereby affecting their metabolism, such as nuclear export or degradation^35^. After X-ray exposure, we isolated RNA from the nucleus, the cytoplasm, and the whole cell lysates of both control and ALKBH5 knockdown cells for RNA sequencing. The results indicated that the distribution patterns of total mRNAs, nuclear mRNAs, and cytoplasmic mRNAs were distinct, and ALKBH5 knockdown influenced the expression of a group of mRNAs within the HEK293T cells (Fig. 3D). Based on the m6A-seq results, we identified the transcripts with increased m6A modifications in ALKBH5 knockdown cells and designated them as ALKBH5 target mRNAs (ALKBH5 targets). Compared to non-ALKBH5 target mRNAs (non-target), knockdown of ALKBH5 significantly altered the subcellular expression of ALKBH5 targets (Fig. 3E). Specifically, the expression of ALKBH5 target genes were upregulated in the whole cell lysates and the cytoplasm compared to non-target genes, while their expression in the nucleus showed no significant difference (Fig. 3E). These results indicate that ALKBH5 likely promotes the degradation of target mRNAs in the cytosol without affecting mRNA metabolism in the nucleus.

### ALKBH5 regulates the stability and expression of CDKN1A and CDKN2B

**Fig. 4:**
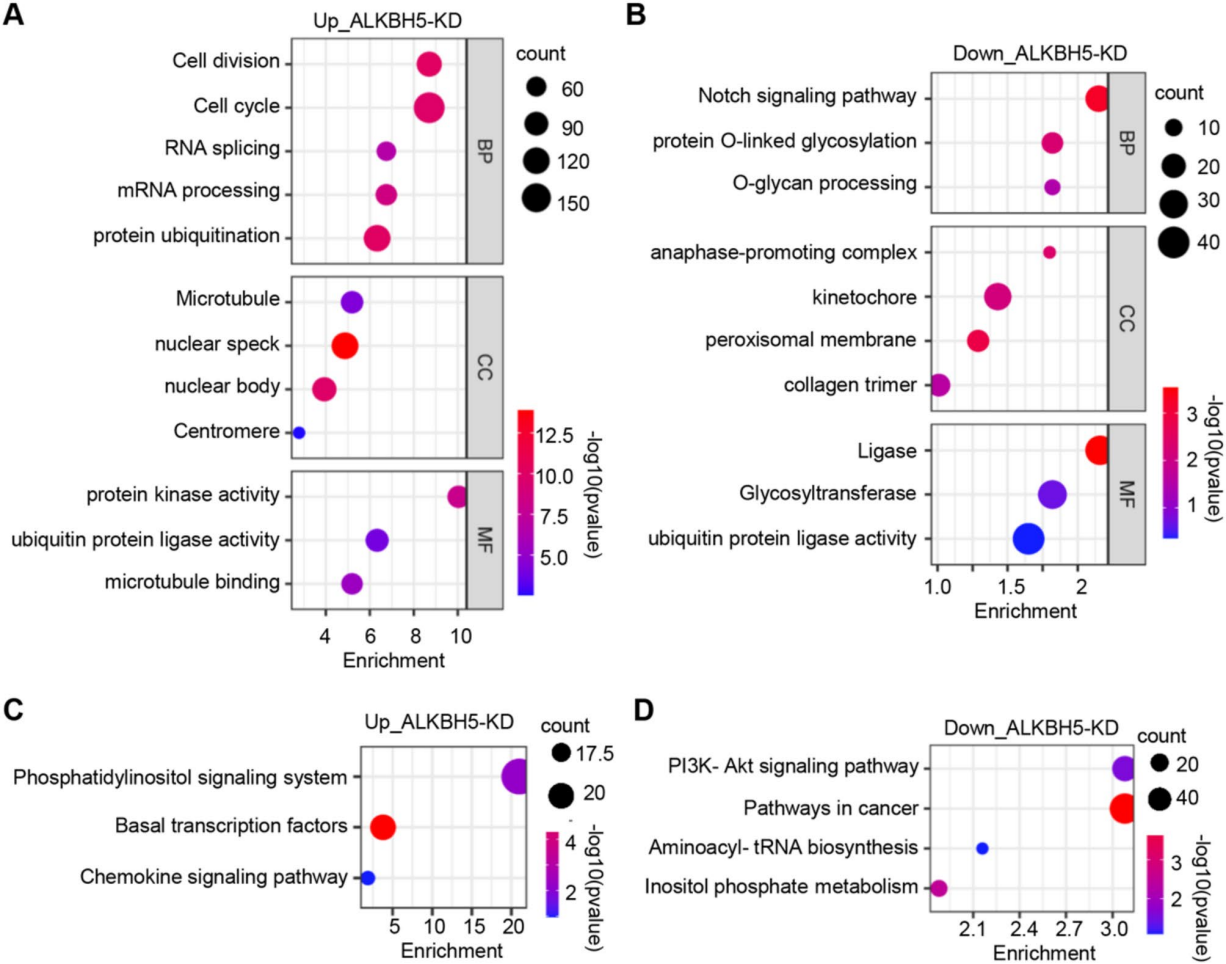
Analysis of ALKBH5-regulated mRNAs. (**A**) Gene ontology analysis of up-regulated ALKBH5 target mRNAs after ALKBH5 knockdown. *BP* biological function, *CC* cellular compartment, *MF* molecular function. (**B**) Gene ontology analysis of down-regulated ALKBH5 target mRNAs after ALKBH5 knockdown. (**C**) KEGG analysis^36–38^ of up-regulated ALKBH5 target mRNAs after ALKBH5 knockdown. (**D**) KEGG analysis of down-regulated ALKBH5 target mRNAs after ALKBH5 knockdown.

To clarify the function of these ALKBH5 target mRNAs upon X-ray exposure, we performed Gene Ontology (GO) enrichment analysis. The results indicated that the upregulated genes in ALKBH5-depleted cells primarily participated in biological processes such as cell cycle regulation (GO: 0007049) and mRNA processing (GO: 0006397) (Fig. 4A). Nuclear-associated components, such as the nuclear speck (GO: 0016607) and body (GO: 0016604), were the most affected cellular components (Fig. 4A). Protein kinase activity (GO: 0004672) and ubiquitin protein ligase activity (GO: 0061630) were mostly enriched molecular functions (Fig. 4A). Additionally, Kyoto Encyclopedia of Genes and Genomes (KEGG) analysis revealed the phosphatidylinositol signaling pathway (HSA: 04070) as significantly enriched (Fig. 4C).

Next, we annotated the function of the down-regulated genes upon ALKBH5 knockdown. They are primarily associated with biological processes such as Notch signaling pathway (GO: 0007219) and protein O-linked glycosylation (GO: 0006493) (Fig. 4B). Changes in cell components were mainly enriched in kinetochore (GO: 0000776) and peroxisomal membrane (GO: 0005778), with molecular functions notably enriched in ligase activity (GO: 0016874) (Fig. 4B). KEGG pathway analysis showed they were primarily enriched in pathways related to cancer (HSA: 05200) and PI3K-Akt signaling (HSA: 04151) (Fig. 4D).

Given that ALKBH5 targeted mRNAs show upregulation after ALKBH5 knockdown (Fig. 3E). We focus on the up-regulated genes for further analysis. One of the enriched group is cell cycle regulation, which is crucial for DNA damage repair. When cells experience severe DNA damage, these checkpoints halt the cell cycle, preventing cell division to provide the necessary time for DNA repair, thereby maintaining genomic stability^39^.

**Fig. 5:**
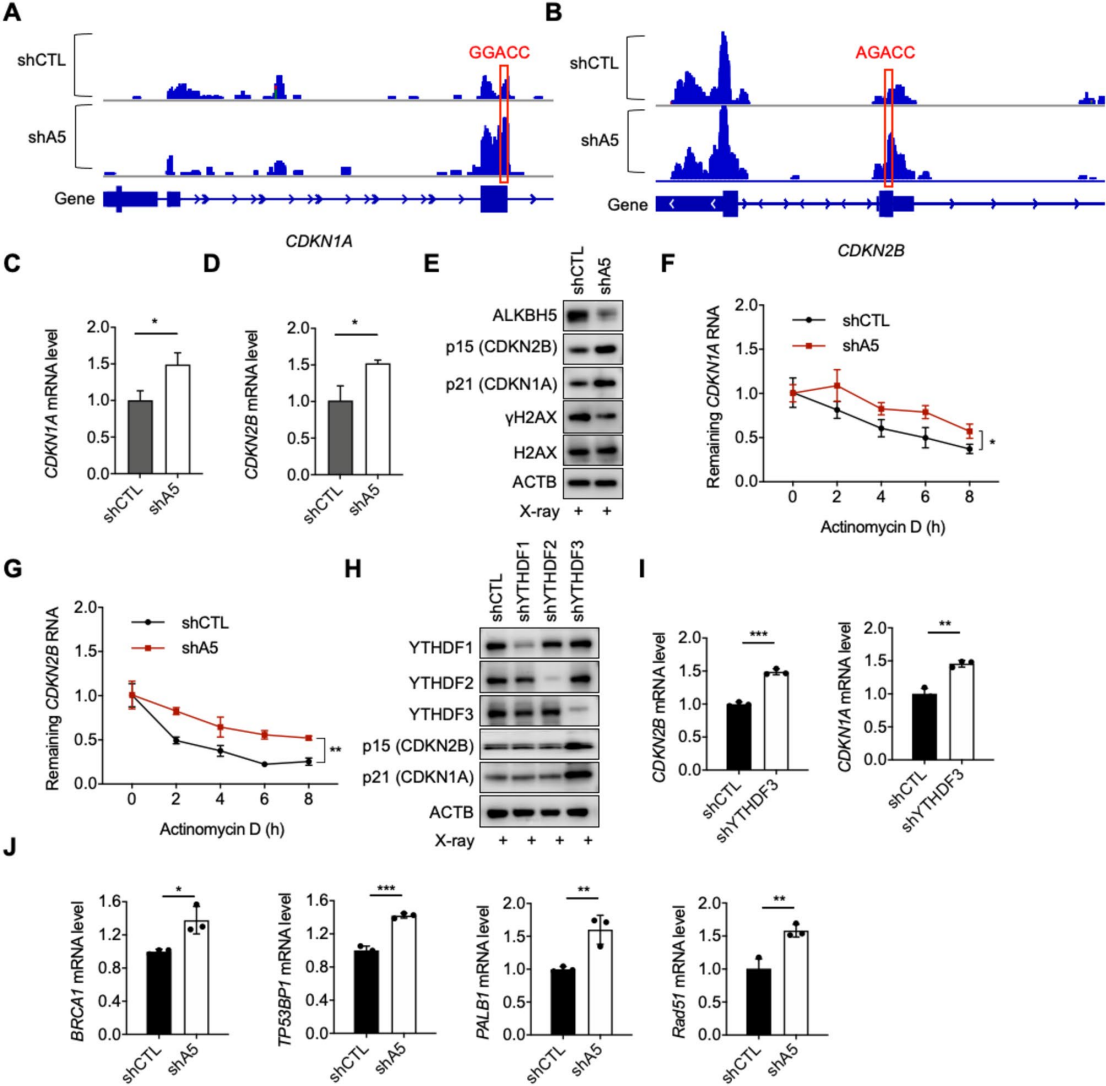
ALKBH5 regulates the stability and expression of CDKN1A and CDKN2B through YTHDF3. (**A**) IGV tracks exhibiting the reads coverage of CDKN1A gene in m6A-seq data from the control and ALKBH5-KD HE293T cells. The RRACH sites located within m6A peaks are marked by red rectangle. (**B**) IGV tracks exhibiting the reads coverage of CDKN2B gene in m6A-seq data from the control and ALKBH5-KD HE293T cells. The RRACH sites located within m6A peaks are marked by red rectangle. (**C**, **D**) The mRNA expression levels of CDKN1A (C) and CDKN2B (D) in the control and ALKBH5 knockdown cells after X-ray exposure. The values obtained are indicated as Mean ± SD. Data were compared statistically using the two-tailed Student’s t-test. (*, *p* < 0.05). (**E**) Immunoblotting of CDKN1A and CDKN2B in the control and ALKBH5 knockdown cells after X-ray exposure. Original blots are presented separately. (**F**, **G**) The mRNA degradation curves of CDKN1A (F) and CDKN2B (G) in the control and ALKBH5 knockdown cells after X-ray exposure. The values obtained are indicated as Mean ± SD. Data were compared statistically using the two-tailed Student’s t-test. (*, *p* < 0.05; **, *p* < 0.01). (**H**) Immunoblotting of CDKN1A and CDKN2B in the control and YTHDF1/2/3 knockdown cells after X-ray exposure. Original blots are presented separately. (**I**) The mRNA level of *CDKN2B*/*CDKN1A* in the control and YTHDF3 knockdown cells following X-ray exposure. (**J**) The mRNA level of DNA factors in HEK293T cells following X-ray exposure.

Among the genes enriched in the cell cycle pathway, the most significantly altered were cyclin-dependent kinase inhibitors (CDKIs) CDKN1A and CDKN2B, which promote cell cycle arrest. CDKN1A encodes the protein p21, a broad-spectrum inhibitor of cyclin/CDK activity, functioning at all stages of the cell cycle^40–43^. CDKN2B encodes the protein p15, which mainly inhibits the binding of the cyclin D-CDK4/6 complex, thereby preventing CDK4/6 activation and affecting G1/S phase progression^44,45^. Visualization of the m6A-seq data using IGV software revealed increased m6A level on CDKN1A and CDKN2B mRNA upon ALKBH5 knockdown (Fig. 5A,B). Furthermore, real-time quantitative PCR results showed that the expression of CDKN1A and CDKN2B were elevated in the ALKBH5 knockdown cells after X-ray exposure (Fig. 5C,D). Immunoblotting also showed upregulation of the proteins expression (Fig. 5E). To assess mRNA stability, actinomycin D was used to inhibit transcription. The results demonstrated that the mRNA degradation rates of CDKN1A and CDKN2B were significantly slower in the ALKBH5 knockdown cells compared to the control cells (Fig. 5F,G). These findings suggest that ALKBH5 knockdown increases m6A modification on CDKN1A and CDKN2B mRNAs, thereby promoting their stability and expression, and contributing to cell cycle regulation.

To investigate the involvement of key m6A readers in this process, we focused on YTHDF1/2/3 family members. The results showed that YTHDF3 knockdown significantly increased both the mRNA level and the protein level of CDKN1A/CDKN2B, resembling the effect of ALKBH5 knockdown (Fig. 5H,I). These findings indicate that YTHDF3 is the reader mediating the expression of CDKN1A/CDKN2B. Moreover, we identified several DNA repair genes, including *BRCA1*, *TP53BP1*, *PALB1* and *Rad51*, as potential ALKBH5 targets from RNA-seq data. RT-qPCR confirmed that the expression of these DNA repair genes significantly upregulated following ALKBH5 knockdown (Fig. 5J), suggesting a direct link between ALKBH5 and DNA repair pathways.

### ALKBH5 depletion increases G2-phase cell proportion and inhibits apoptosis

**Fig. 6:**
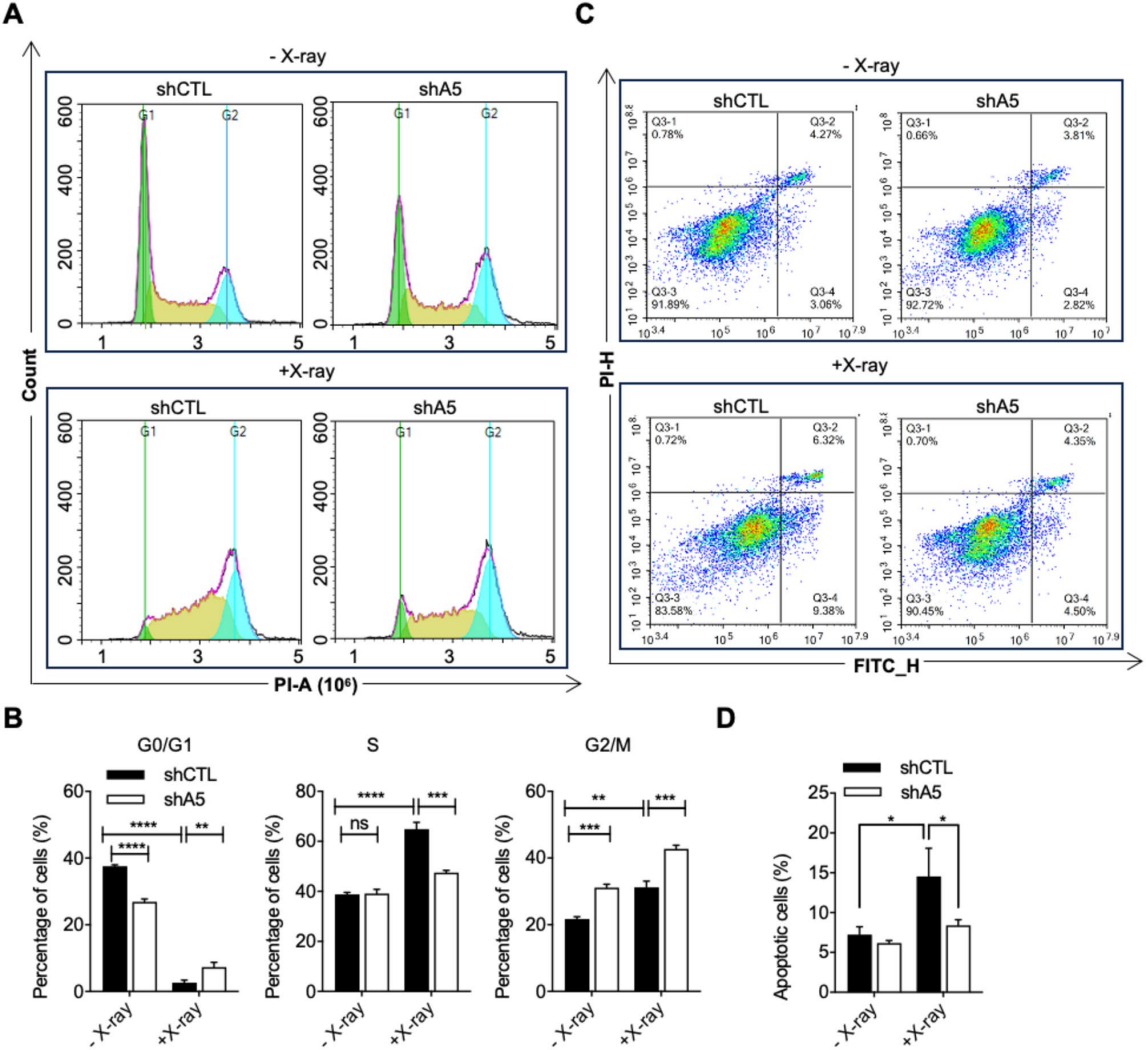
Knockdown of ALKBH5 increases the proportion of G2 phase cells and inhibits cell apoptosis. (**A**) The cell cycle of control and ALKBH5 knockdown cells before and after X-ray exposure as determined by flow cytometry. (**B**) Statistical analysis of the percentage of cells in G0/G1, S, and G2/M phases before and after X-ray exposure in the control and ALKBH5 knockdown cells. The values are indicated as Mean ± SD. Data were compared statistically using the two-tailed Student’s t-test. (ns, not significant; **, *p* < 0.01; ***, *p* < 0.001; ****, *p* < 0.0001). (**C**) Annexin-V-positive cells of control and ALKBH5 knockdown cells before and after X-ray exposure as determined by flow cytometry. (**D**) Statistical analysis of the percentage of apoptotic cells in control and ALKBH5 knockdown cells. The values are indicated as Mean ± SD. Data were compared statistically using the two-tailed Student’s t-test. (*, *p* < 0.05; **, *p* < 0.01).

Having confirmed the regulation of CDKN1A and CDKN2B by ALKBH5, we next evaluated the impact of ALKBH5 on the cell cycle using flow cytometry. Cells were collected 12 h after exposure to 10 Gy X-rays. The results showed a significant induction in the proportion of G2 phase cells post X-ray exposure (Fig. 6A,B). This indicates that cells arrested in G2 phase following X-ray-induced DNA damage. ALKBH5 knockdown significantly increased the proportion of cells in the G2-phase (Fig. 6A,B), suggesting that ALKBH5 knockdown can markedly inhibit cell cycle progression, providing time for DNA repair and preventing the transmission of DNA damage. Additionally, cells apoptosis was assessed 24 h after X-ray irradiation. The results indicated that ALKBH5 knockdown significantly reduced the proportion of cell apoptosis after X-ray irradiation (Fig. 6C,D).

**Fig. 7:**
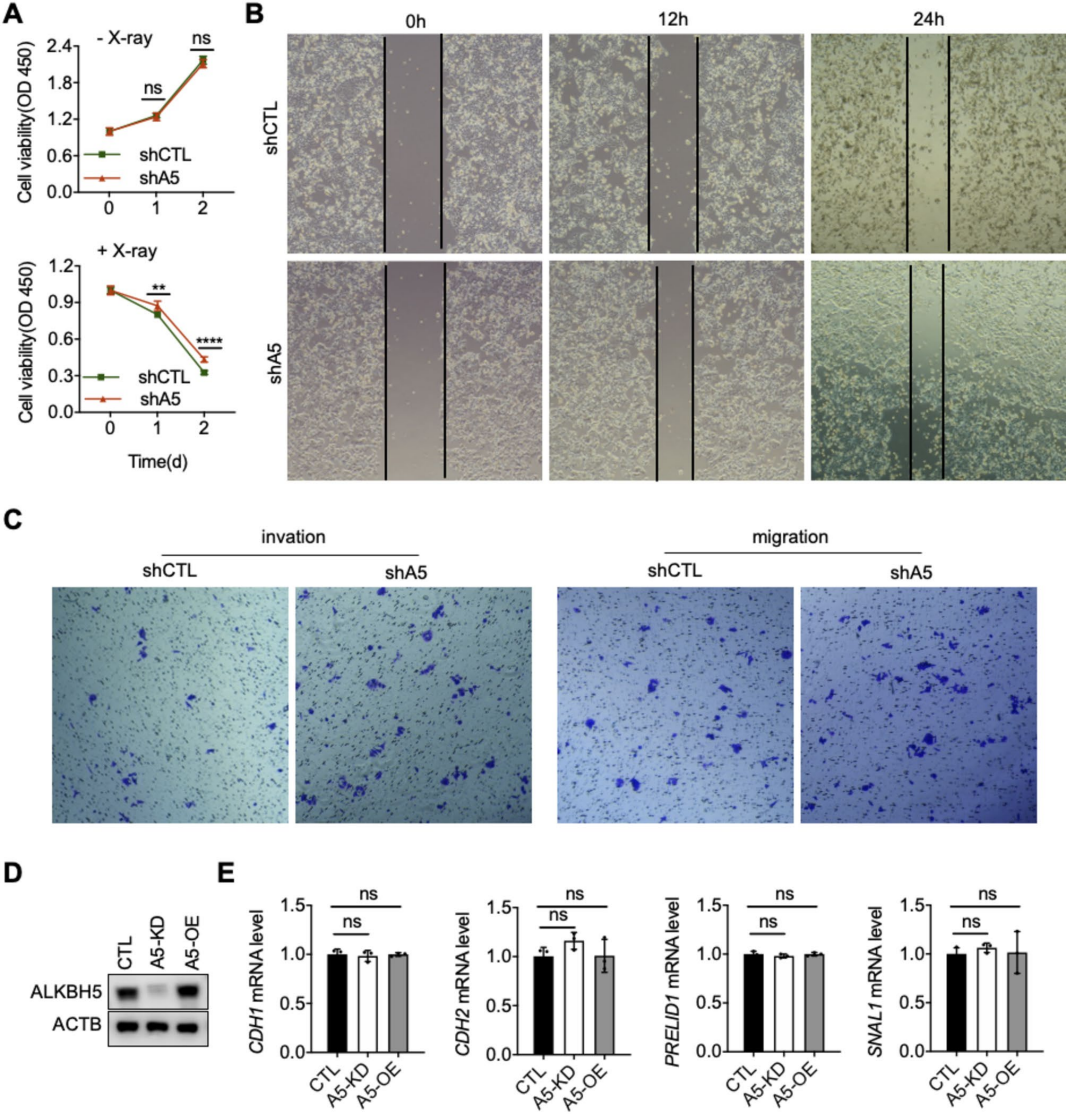
ALKBH5 promotes the malignant phenotype of HCT116 cells (10 Gy). (**A**) Cell viability of HCT116 cells with or without X-ray treatment (*n* = 5, *t*-test). (**B**) Representative image of wound healing assay of HCT116 cells. (**C**) Representative image of transwell assays of HCT116 cells. (**D**) Validation of A5 knockdown and overexpression. Original blots are presented separately. (**E**) The mRNA level of EMT markers in HCT116 cells following X-ray exposure. **P < 0.01, ***P < 0.001, ****P < 0.0001.

We then examined the impact of ALKBH5 on the proliferation, migration, and invasion abilities of the HCT116 cells. The results showed that these abilities were enhanced in HCT116 cells upon ALKBH5 knockdown (Fig. 7A–C). However, ALKBH5 had no effect on the expression of epithelial-mesenchymal transition (EMT) markers (Fig. 7D,E). These findings suggest that ALKBH5 inhibits the malignant phenotype of HCT116 cells, potentially mediated through mechanisms independent of EMT. In conclusion, after X-ray-induced DNA damage, ALKBH5 knockdown promotes the m6A deposit on cyclin dependent kinase inhibitors, enhancing their stability and expression. This leads to cell cycle G2/M phase arrest, facilitating DNA damage repair, and reducing apoptosis.

## Discussion

**Fig. 8:**
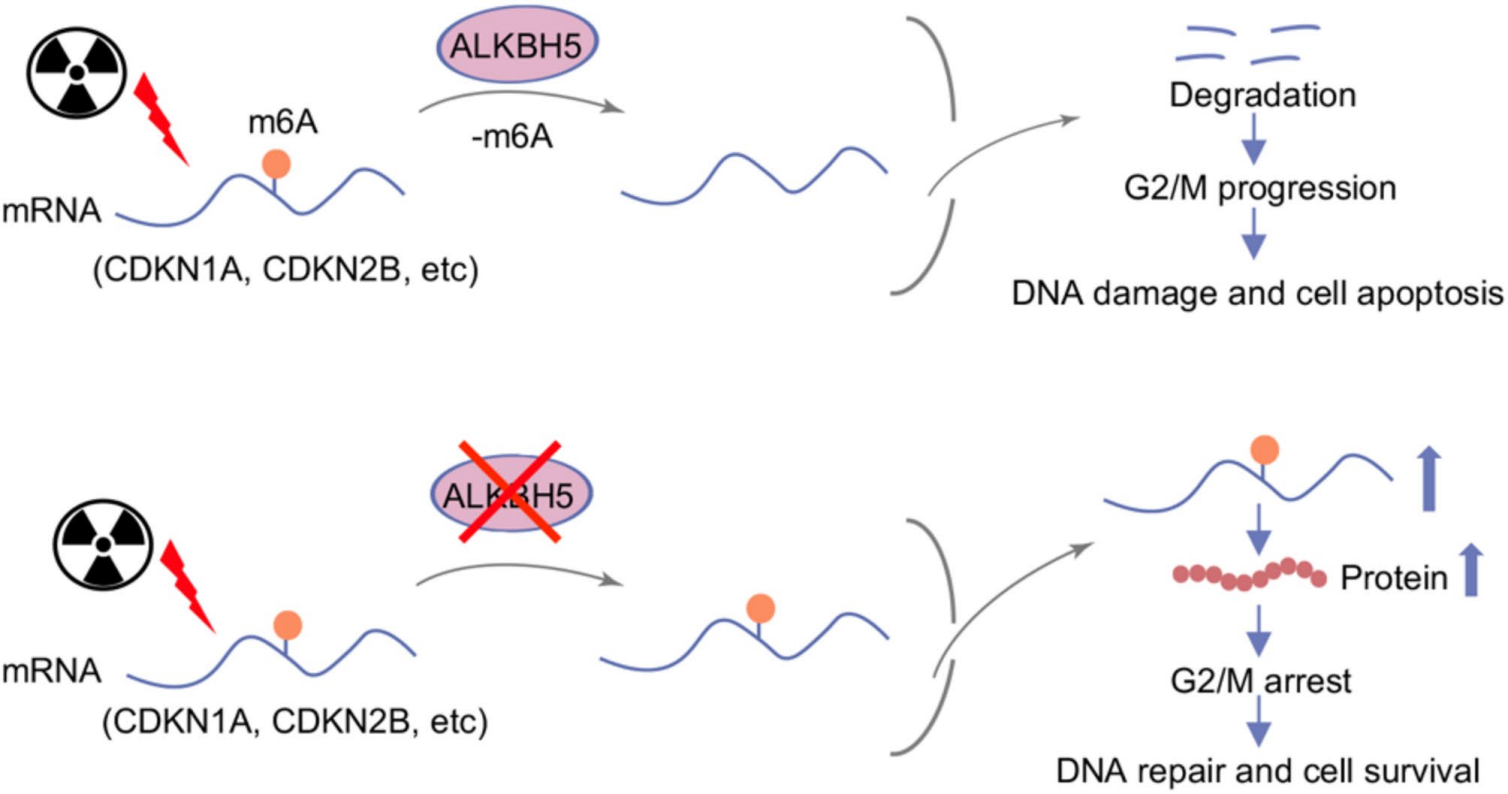
Schematic diagram illustrating the role of ALKBH5 in X-ray-induced DDR.

Increasing evidence highlights the function of RNA m6A machinery in DDR^28,46^. In this study, we demonstrated a crucial role of the m6A ‘eraser’ ALKBH5 in X-ray-induced DDR. Knockdown of ALKBH5 significantly reduced DNA damage after X-ray exposure and promoted the repair of DNA double-strand breaks through both HR and NHEJ pathways. Mechanistically, during the DDR triggered by X-rays, ALKBH5 knockdown enhanced the m6A modification in cyclin dependent kinase inhibitors mRNAs, improving their stability and expression. As a result, this facilitated cell cycle arrest, providing ample time for DNA repair and subsequently reducing apoptosis (Fig. 8). In conclusion, our findings suggest that ALKBH5 post-transcriptionally regulates cell cycle checkpoint in the DDR caused by X-ray irradiation.

DDR is a signaling cascade that detects, amplifies, and transmits damage signals, involving key processes such as cell cycle regulation, DNA repair, and apoptosis^7,8,47^. The cell cycle checkpoint is a crucial mechanism for monitoring and repairing DNA damage during cell cycle^9^. When cells experience severe DNA damage, checkpoints halt cell cycle progression to allow time for DNA repair, thereby maintaining genomic stability^9,10^. Our results indicate that X-ray irradiation causes a significant accumulation of cells in the G2 phase, while knockdown of ALKBH5 significantly increases the proportion of cells in the G2 phase. These data indicate that ALKBH5 knockdown can markedly halt cell division, allowing time for DNA damage repair and preventing the transmission of DNA damage. Notably, after X-ray exposure, the proportion of apoptotic cells is significantly lower in ALKBH5 knockdown cells, which aligns with the observed reduction in DNA damage and enhanced DSB repair following ALKBH5 knockdown. Moreover, ALKBH5 knockdown upregulates key DNA repair factors, including *BRCA1*, *TP53BP1*, *PALB1* and *RAD51*, suggesting a direct link between ALKBH5 and DNA repair pathways. Collectively, during X-ray-induced DDR, ALKBH5 knockdown facilitates DNA repair, ultimately promoting the restoration of normal cell cycle progression and reducing apoptosis.

ALKBH5 is one of the RNA m6A ‘eraser’ in mammals^35^. It catalyzes the demethylation of RNAs, primarily mRNAs, thereby influencing metabolism such as nuclear export and RNA decay, and ultimately regulating gene expression^35^. Through m6A RNA sequencing, we further confirmed that knocking down ALKBH5 significantly increases the RNA m6A deposits following X-ray treatment. Interestingly, RNA sequencing revealed that after ALKBH5 knockdown, the average expression of ALKBH5 target genes, exhibiting increased m6A abundance, was higher in both the whole cell and the cytoplasm compared to non-target genes. However, there was no significant difference in the average expression within the nucleus. These results indicate a role of ALKBH5 in promoting mature mRNA decay, while the detailed mechanism warrants further investigation. Given the well-established roles of YTHDF1/2/3 in the m6A regulation, we investigated them as mediators of ALKBH5-regulated mRNA stability. YTHDF3 knockdown significantly increased the expression of CDKN1A/CDKN2B, resembling the effect of ALKBH5 knockdown. These findings indicate that YTHDF3 is essential for the regulation of mRNA stability by ALKBH5.

GO enrichment analysis of upregulated ALKBH5 target genes after X-ray treatment shows that these genes mainly regulate the cell cycle and mRNA processing. Among the enriched cell cycle pathway, the most significantly affected genes are CDKN1A and CDKN2B. These two genes belong to the cyclin-dependent kinase inhibitors, which promote cell cycle arrest. CDKN1A encodes the protein p21, which broadly inhibits cyclin/CDK activity and functions across all stages of the cell cycle^40–42^. CDKN2B encodes p15, which mainly inhibits the binding of the cyclin D-CDK4/6 complex, thereby preventing CDK4/6 activation and affecting G1 phase progression^44,45^. Consistent with increased expression of CDKN1A and CDKN2B, we observed increased G2/M phase arrest in ALKBH5 knockdown cells. In line with our data, previous studies have shown that ALKBH5-mediated m6A RNA demethylation specifically affects the expression of CDK inhibitors in several cell types such as γδT cell progenitors and tumor cells^48^. In our study, we confirmed that following X-ray exposure, ALKBH5 knockdown increases the m6A modification of CDKN1A and CDKN2B, significantly reducing their degradation rate and, consequently, increasing their mRNAs expression. Collectively, these results indicate that ALKBH5 promotes cell cycle progression under various conditions by inhibiting the expression of CDK inhibitors.

Several studies have reported that ALKBH5 knockdown tends to induce G1 arrest in various cell lines^49–52^. The discrepancy in ALKBH5’s role in cell cycle regulation can be attributed to differences in cell context, DNA damage type, functional redundancy, and experimental design. Cell lines with distinct genetic backgrounds may exhibit varied responses to ALKBH5 knockdown due to unique signaling pathways. Additionally, while X-ray exposure in our study induces G2/M arrest, other DNA damage types may lead to different outcomes. Functional redundancy with other m6A demethylases or compensatory mechanisms could also shift the arrest phase. A systematic comparison across different conditions is essential to fully elucidate ALKBH5’s role in DNA damage repair and cell cycle regulation.

ALKBH5 is an RNA demethylase that regulates gene expression by removing m6A modifications. Its expression is closely associated with tumor proliferation, invasion, and metastasis in various cancer types. In non-small cell lung cancer (NSCLC), ALKBH5 has been found to inhibit TGF-β-induced epithelial-mesenchymal transition (EMT) by regulating the TGF-β/SMAD signaling pathway, thereby suppressing tumor invasion and metastasis^53^. In pancreatic cancer (PC), ALKBH5 enhances the stability of PER1 by removing the m6A modification on PER1 mRNA, thereby activating the ATM-CHK2-P53/CDC25C signaling pathway to inhibit tumor cell growth^54^. Our data confirmed that ALKBH5 knockdown promote proliferation of HCT116 cells and induces chemoresistance, and enhances its malignant phenotype. ALKBH5 functions as a tumor suppressor, which is consistent with previously published studies^55,56^. Meanwhile, it does not significantly affect the expression of EMT markers such as E-cadherin, N-cadherin, Vimentin, and Snail. This suggests that the pro-proliferative and chemoresistance-inducing effects of ALKBH5 knockdown in HCT116 cells may be mediated through mechanisms independent of EMT. Its effects may be mediated through alternative pathways, which needs further exploration.

Overall, our study reveals a novel role of ALKBH5 in the X-ray-induced DDR. ALKBH5 depletion affects the stability and expression of genes related to DDR, enabling precise and efficient control of protein production during the stress response. This also provides new insights into the involvement of m6A modifications in the DDR, offering potential strategies for DNA damage-based cancer treatment.

## Materials and methods

### Cell culture and treatment

HEK293T cells are obtained from American Type Culture Collection (ATCC) and cultured in Dulbecco’s Modified Eagle Medium (DMEM) (Gibco) with 10% fetal bovine serum (FBS) (Gibco) and 1% penicillin-streptomycin (Sangon Biotech) at 37 °C and 5% CO2. For X-ray irradiation treatment, cells were exposed to 10 Gy X-ray and harvested after 1, 12 and 24 h, respectively.

### Cell culture and treatment

HEK293T cells are obtained from ATCC and cultured in Dulbecco’s Modified Eagle Medium (DMEM) (Gibco) with 10% fetal bovine serum (FBS) (Gibco) and 1% penicillin-streptomycin (Sangon Biotech) at 37 °C and 5% CO2. For X-ray irradiation treatment, cells were exposed to 10 Gy X-ray and harvested after 1 h, 12 h and 24 h respectively.

### Immunofluorescence

Cells were seeded at approximately 30% density in a 12-well plate containing glass coverslips. After 24 h of adhesion, the cells were fixed for 15 min using 4% polyformaldehyde solution and then permeabilized with 0.1% Triton X-100 for 15 min. After blocking with goat serum for 1 h, cells were then exposed to a γH2AX antibody (diluted at 1:100 in PBS) at room temperature for 2 h. Following the primary antibody incubation, cells were incubated with a fluorescence-labeled anti-mouse IgG (1:1000), stained with DAPI and mounted with an anti-fade mounting medium. Images were taken using the Nikon A1R Confocal Microscope.

### Immunoblotting

Cells were collected using RIPA lysis buffer (200 µL/10 cm²). Proteins were separated using sodium dodecyl sulfate-polyacrylamide gel electrophoresis (SDS-PAGE) of appropriate concentration based on the molecular weight of the target protein, and then transferred onto a PVDF membrane. The membrane was blocked and incubated with primary antibodies, followed by secondary antibodies, and detected using a chemiluminescence detection system (Amersham Imager).

### Comet assay

Prepare a cell suspension, and mix 10 µL of the suspension with 75 µL of 0.7% low-melting-point agarose. Gently spread the mixture on a slide, flatten it with a coverslip, and place on ice until the agarose solidifies. Remove the coverslip and immerse the slide in pre-cooled lysis buffer at 4 °C for 1 h. After lysis, incubate the slide in neutral electrophoresis solution (pH 8.0) for another 30 min. Run electrophoresis at a voltage of 1 V/cm for 15 min. Neutralize the slide in Tris-HCl buffer three times, 5 min each time. Stain the slide with propidium iodide (PI) in the dark for 10 min, mount the slide. Images were taken using a fluorescence microscope. ImageJ was used to quantify the comet tail moment.

### Cell cycle analysis

Cells were collected 12 h later post X-ray exposure and washed twice with pre-chilled PBS. A volume of 1 mL of pre-chilled 70% ethanol was added to the cell pellet, and the mixture was gently mixed before fixing at 4 °C for 30 min. The cells were centrifuged at 800 rpm for 3 min, followed by two washes with pre-chilled PBS. After removing the supernatant, 0.5 mL of PI staining solution was added to each cell sample. The cell pellet was resuspended and incubated at 37 °C in the dark for 30 min. Red fluorescence was detected using a flow cytometer.

### Apoptosis assay

At 24 h post-X-ray irradiation, cells were collected into centrifuge tubes and washed twice with pre-chilled PBS. After discarding the supernatant, 100 µL of 1× Binding Buffer was added to resuspend the cells. Add 5 µL of Annexin V-FITC and 10 µL of PI, vortex the mixture gently and incubate in the dark at room temperature for 15 min. Next, add 400 µL of 1× Binding Buffer and place the samples on ice. Samples were analyzed by flow cytometry within 1 h.

### HR and NHEJ reporter assay

For the HR experiment, cells were co-transfected with the DR-GFP reporter plasmid and the I-SceI plasmid. Transient expression of I-SceI endonuclease generates DNA-DSBs at the integrated GFP gene sequences, and successful repair through HR results in GFP expression^57^. NHEJ reporter utilizes the two broken ends of a GFP gene separated by an adenovirus exon digested with HindIII^58^. The NHEJ-GFP reporter plasmid was digested with HindIII restriction enzyme for 6 h and linearized DNA was then transfected into HEK293T cells. For both experiments, mCherry was co-transfected to serve as a transfection control. After 48 h incubation, the proportion of GFP-positive cells among RFP-positive cells was assessed using flow cytometry, and the repair efficiency was calculated.

### Lentivirus generation and cell infection

For shRNA knockdown experiments, specific shRNA oligos targeting ALKBH5 were annealed and cloned to the lentiviral vector pLKO.1. To package lentivirus, HEK293T cells were co-transfected with the shRNA plasmid, along with packaging plasmids (including psPAX2 and pMD2.G) using a transfection reagent. After 48–72 h incubation, the supernatant containing the lentivirus was collected, filtered. For cell infection, HEK293T cells were seeded in a suitable density, and the lentiviral supernatant was added to the culture medium. After 24 h, fresh culture medium was replaced and puromycin was added to eliminate uninfected cells. The shRNAs used in this study are listed in Table S2.

### RNA isolation, cDNA synthesis and quantitative RT–PCR

RNA was isolated from HEK293T cells using TRIzol reagent (Invitrogen). cDNA synthesis was carried out with the Evo M-MLV Master Mix (Accurate Biology) using random primers. Perform real-time quantitative PCR using the LightCycler480 (Roche). Relative gene expression was normalized to β-ACTIN or GAPDH expression and determined using the 2^(−ΔΔCT)^ method. The RT-qPCR primers used in this study are listed in Table S2.

### RNA-seq

RNA was isolated using Trizol Reagent (Invitrogen) and mRNA was enriched using Dynabeads Oligo(dT)25 (Invitrogen). Fragmentation reagents were added to break the mRNA into fragments approximately 100–200 nucleotides. These fragments were then collected for library construction and high-throughput sequencing.

### m6A-seq

For m6A library construction, fragmented RNA was mixed with Dynabeads G coated with m6A antibody and incubated at 4 °C for 4 h. The immunoprecipitated complexes were digested with proteinase K and the RNA was precipitated with ethanol. The purified RNA was subsequently used for library construction and high-throughput sequencing.

### RNA stability assay

Cells were treated with 10 µg/mL Actinomycin D to inhibit transcription. Cell samples were collected at 0, 2, 4, 6, and 8 h post-treatment to extract RNA and perform real-time quantitative PCR. Calculate the RNA abundance of the target gene at each specified time point as a percentage of the RNA abundance at 0 h, and plot the RNA stability curve.

### Wound healing assay

Mark the bottom of a 6-well plate with three horizontal reference lines using a marker and ruler. Seed cells at a density of 5 × 10⁵ cells per well, ensuring even distribution. Once the cells reach confluence, use a 200 µL pipette tip to make two vertical scratches intersecting the reference lines, creating fixed detection points. Gently wash the cells 3 times with PBS to remove scratched cells. Take photos of the scratch at various magnifications as the 0-hour control, then incubate the cells at 37 °C with 5% CO₂. At the required time points, observe the width of the scratch and take additional photos.

### Transwell assay

Observe the cell growth, and when cells are in good condition, thaw the Matrigel on ice. Under 4 °C, dilute the Matrigel with serum-free medium or PBS at a 1:8 ratio, mix until uniform, and add 60 µL vertically to the bottom of the Transwell chamber. Incubate at 37 °C for 2 h to allow the Matrigel to polymerize. Aspirate unbound Matrigel, add 100 µL serum-free medium to each well, and incubate for 30 min to hydrate. Remove the liquid and check for any passage through the chamber. When cells reach logarithmic growth, centrifuge, discard the supernatant, wash with PBS, and prepare a suspension of 3 × 10⁵ cells/100 µL in serum-free medium. Add 500 µL medium with 20% FBS to the lower chamber, insert the Transwell into the 24-well plate, and add the cell suspension to the upper chamber. Compared to the invasion assay, the migration assay uses one less layer of Matrigel. Incubate for 48 h. After incubation, remove the insert, aspirate the medium, and gently wipe the Matrigel and cells with a PBS-wet cotton swab. Fix with 600 µL 4% paraformaldehyde for 20 min. Discard the fixative, wash with PBS, stain with crystal violet for 15 min, then wash again and take images under a microscope.

### Sequencing data analysis

Download the human reference genome file (version hg38) and the corresponding annotation files. Use FastQC to assess the quality of the raw high-throughput sequencing data and process the data with fastp. Align the sequencing reads to the reference genome (hg38) using HISAT2, then use Samtools to sort and convert the data into BAM file format. Gene quantification was performed with StringTie. The m6A-enriched regions within each m6A-immunoprecipitation sample were identified using MACS2 software. The enriched m6A motifs were analyzed using MEME. DAVID was used for GO functional enrichment analysis.

### Statistical analysis

Statistical analysis was carried out using Graphpad Prism 9. Statistical significance was assessed using the Student’s t-test and Mann-Whitney U test, depending on the data type. All data comparisons were performed with two-tailed tests, and *p* < 0.05 was considered statistically significant.

## Electronic supplementary material

Below is the link to the electronic supplementary material.

Supplementary Material 1

Supplementary Material 2

## Data Availability

Sequencing data have been deposited into the Gene Expression Omnibus (GEO) under the accession number GEO: GSE281853 (https://www.ncbi.nlm.nih.gov/geo/query/acc.cgi? acc=GSE281853). The secure token is avojwywyppmtrmx. Please contact to Jingyu Hou(jingyuhou@zju.edu.cn) if someone wants to request the data from this study.
