# Supplementary material for: RNA demethylase ALKBH5 regulates cell cycle progression in DNA damage response
Source: Sci Rep. 2025 May 8;15:16059. doi: 10.1038/s41598-025-01207-8 (PMC12062394; doi:10.1038/s41598-025-01207-8)
Supplement: Supplementary file 2 — Supplementary Material 2 [file 41598_2025_1207_MOESM2_ESM.docx]

**RNA demethylase ALKBH5 regulates cell cycle progression in DNA damage response**

Bo Gao ^1,#^, Haitao Pan ^1,#^, Xiaoling Zhou ^2^, Lei Yu ^2^, Yunyi Gao ^2^, Tao Zhang ^1,^* and Xiangwei Gao ^2,^*, Jingyu Hou ^2,#,^*.

Original blots
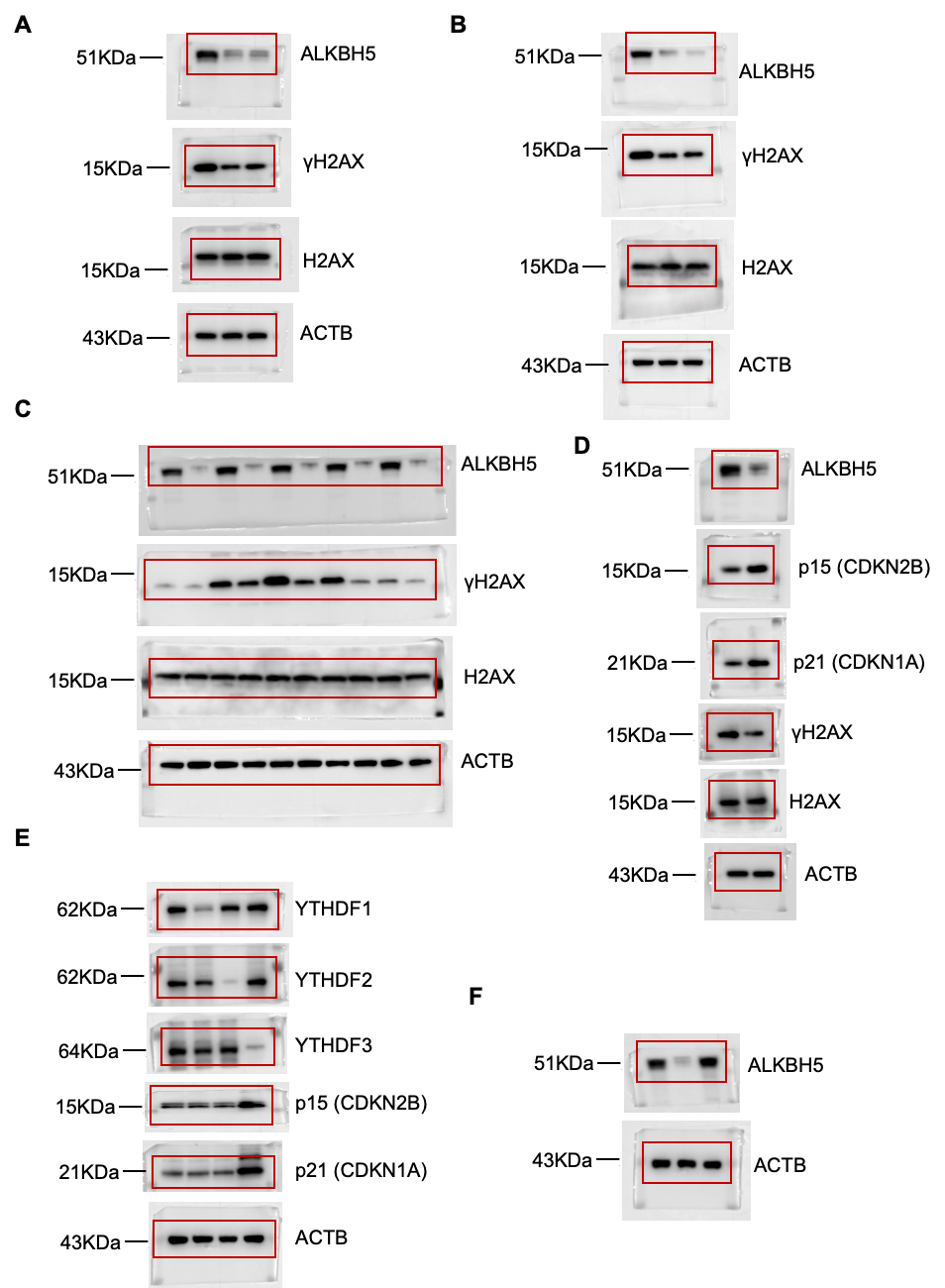


**Original blots in the main manuscript.**

(A) Original blots of Figure 1A.

(B) Original blots of Figure 1B.

(C) Original blots of Figure 1G.

(D) Original blots of Figure 5E.

(E) Original blots of Figure 5H.

(F) Original blots of Figure 7D.
